# YTH domain family protein 3 accelerates non-small cell lung cancer immune evasion through targeting CD8^+^ T lymphocytes

**DOI:** 10.1038/s41420-024-02084-2

**Published:** 2024-07-11

**Authors:** Yisheng Luo, Chao Zeng, Zezhong Ouyang, Wenbin Zhu, Jiazhi Wang, Zhiyin Chen, Chunyang Xiao, Guodong Wu, Liang Li, Youhui Qian, Xin Chen, Yuchen Liu, Hao Wu

**Affiliations:** 1 Department of Thoracic Surgery, The First Affiliated Hospital of Shenzhen University, Shenzhen Second People’s Hospital, Shenzhen, 518000 Guangdong Province China; 2 Department of Respiratory and Critical Care Medicine, Peking University Shenzhen Hospital, Shenzhen, 518000 Guangdong Province China; 3 National Regional Key Technology Engineering Laboratory for Medical Ultrasound, Guangdong Key Laboratory for Biomedical Measurements and Ultrasound Imaging, School of Biomedical Engineering, Shenzhen University Medical School, Shenzhen University, Shenzhen, 518000 Guangdong Province China; 4 Guangdong Provincial Key Laboratory of Systems Biology and Synthetic Biology for Urogenital Tumors, The First Affiliated Hospital of Shenzhen University, Shenzhen Second People’s Hospital (Shenzhen Institute of Translational Medicine), Shenzhen, 518000 Guangdong Province China

**Keywords:** Immune evasion, Cancer microenvironment

## Abstract

Immune evasion is one of the critical hallmarks of malignant tumors, especially non-small cell lung cancer (NSCLC). Emerging findings have illustrated the roles of N^6^-methyladenosine (m^6^A) on NSCLC immune evasion. Here, this study investigated the function and underlying mechanism of m^6^A reader YTH domain family protein 3 (YTHDF3) on NSCLC immune evasion. YTHDF3 was found to be highly expressed in NSCLC tissue and act as an independent prognostic factor for overall survival. Functionally, up-regulation of YTHDF3 impaired the CD8^+^ T antitumor activity to deteriorate NSCLC immune evasion, while YTHDF3 silencing recovered the CD8^+^ T antitumor activity to inhibit immune evasion. Besides, YTHDF3 up-regulation reduced the apoptosis of NSCLC cells. Mechanistically, PD-L1 acted as the downstream target for YTHDF3, and YTHDF3 could upregulate the transcription stability of PD-L1 mRNA. Overall, YTHDF3 targeted PD-L1 to promote NSCLC immune evasion partially through escaping effector cell cytotoxicity CD8^+^ T mediated killing and antitumor immunity. In summary, this study provides an essential insight for m^6^A modification on CD8^+^ T cell-mediated antitumor immunity in NSCLC, which might inspire an innovation for lung cancer tumor immunotherapy.

## Introduction

Non-small-cell lung cancer (NSCLC) accounts for a large proportion (80–85%) of the whole lung cancer, which is the most commonly diagnosed malignancy and remains the leading cause of tumor-death worldwide [1, 2]. The 5-year survival rate of NSCLC approximately maintain at only 20% [3, 4]. Clinically, cancer immunotherapy could restore or enhance the effector function of CD8^+^ T cells or other immune cells. Therefore, a better understanding for NSCLC immunotherapy is of significant importance.

Immune evasion acts as an important hallmark of malignant tumors, which refers to the phenomenon that cancer cells evasion the recognition and killing of the immune cells [5]. Immune evasion processes continue to evolve in NSCLC invasive stage and are associated with inter/intra-tumor heterogeneity, which contributes to immunotherapy of checkpoint blockade (ICB) [6, 7]. Tumor immune microenvironment (TME) is deeply correlated to the tumor immunotherapy efficacy, whose characteristics could significantly affect the tumor progression and metastasis [8]. TME is crucial for the development of NSCLC, in which NSCLC cells interacted with immune cells (T cells, B cells) to facilitate immune evasion. For example, in NSCLC, ILT4 overexpression suppresses the tumor immunity by impairing T cell response and recruiting M2-like TAMs, preventing immunosuppression and enhancing the efficacy of PD-L1 inhibitor in EGFR wild-type NSCLC [9].

Programmed cell death protein ligands-1 (PD-L1) is one of the PD-1 ligands, which has been shown to be a valuable biomarker for the tumor prognosis [10]. PD-L1 expression is mainly expressed in cancer cells, antigen-presenting cells (APCs) or tumor-infiltrating cells in many cancers [11]. In NSCLC, the functions of PD-L1 have been diffusely reported. For example, PD-L1 expression is regulated by oncogenic drivers, e.g. epidermal growth factor receptor (EGFR) or anaplastic lymphoma kinase (ALK) in NSCLC [12]. Besides, PD-L1 expression is correlated to shorter survival in advanced/metastatic NSCLC [13]. Thus, PD-L1 plays an essential role in tumor immune evasion.

N^6^-methyladenosine (m^6^A) has been reported to be abnormally expressed in numerous cancers, including NSCLC. In this study, this research investigated the role of YTHDF3 on NSCLC immune evasion via m^6^A modification-dependent manner. YTHDF3 could accelerate the immune evasion partially through escaping effector cell cytotoxicity CD8^+^ T mediated killing and antitumor immunity. Mechanistically, YTHDF3 could upregulate the transcription stability of PD-L1 mRNA. Overall, the study provided an essential insight for epigenetics m^6^A modification and CD8^+^ T cell-mediated antitumor immunity in NSCLC.

## Results

### YTHDF3 highly expressed in NSCLC tissue and acted as an independent prognostic factor for overall survival

**Fig. 1:**
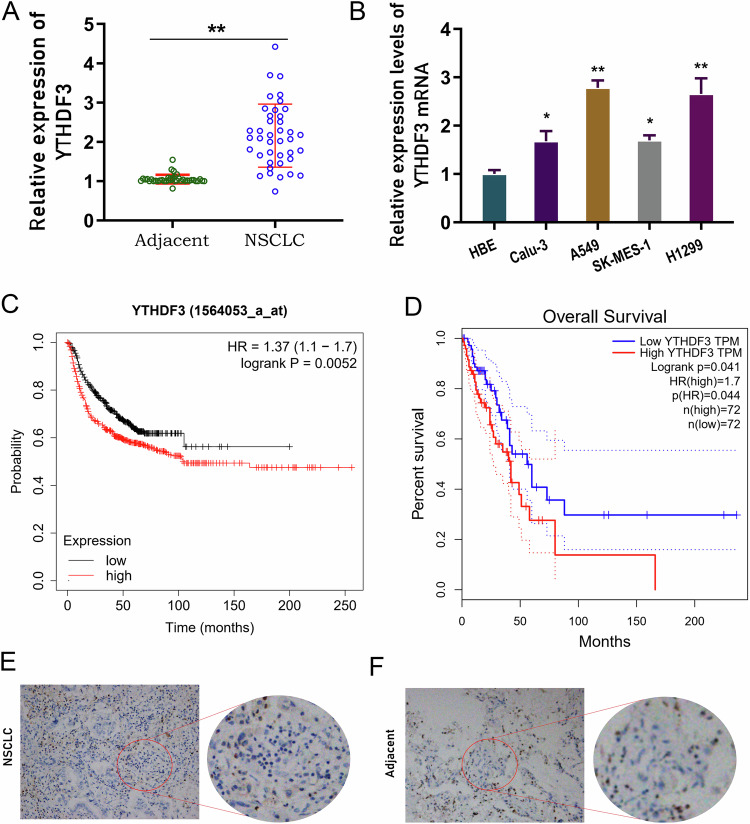
YTHDF3 highly expressed in NSCLC tissue and acted as an independent prognostic factor for overall survival. **A** YTHDF3 level was detected in enrolled cancer samples in NSCLC tissue as comparing with normal tissue. **B** RT-PCR analysis was performed to detect the YTHDF3 level in NSCLC cell lines (Calu-3, A549, SK-MES-1, H1299) and control cells (human normal bronchial epithelial cell, HBE). **C**, **D** The overall survival assessment for lung cancer was determined by in silico predictive analysis Kaplan Meier-plotter (http://kmplot.com/analysis/) and GEPIA (http://gepia.cancer-pku.cn/index.html). **E**, **F** Immunohistochemical analysis was performed to detect the YTHDF3 in the tumor tissue as comparing with adjacent normal tissue. *p < 0.05; **p < 0.01.

Firstly, the clinical function of YTHDF3 in NSCLC was investigated. In the enrolled cancer samples, YTHDF3 highly expressed in NSCLC tissue as comparing with normal tissue (Fig. 1A). In the NSCLC cells (Calu-3, A549, SK-MES-1, H1299), YTHDF3 was also highly expressed as comparing with normal cells (HBE) (Fig. 1B). Overall survival analysis revealed that high-expression YTHDF3 indicated the poor prognosis for NSCLC patients, acting as an independent prognostic factor for NSCLC overall survival (Fig. 1C, D). In the NSCLC tissue, immunohistochemical analysis revealed that YTHDF3 was highly expressed in the tumor tissue as comparing with adjacent normal tissue (Fig. 1E, F). Overall, these findings revealed that YTHDF3 highly expressed in NSCLC tissue and acted as an independent prognostic factor for overall survival.

### YTHDF3 silencing recovered CD8^+^ T antitumor activity to inhibit NSCLC immune evasion

**Fig. 2:**
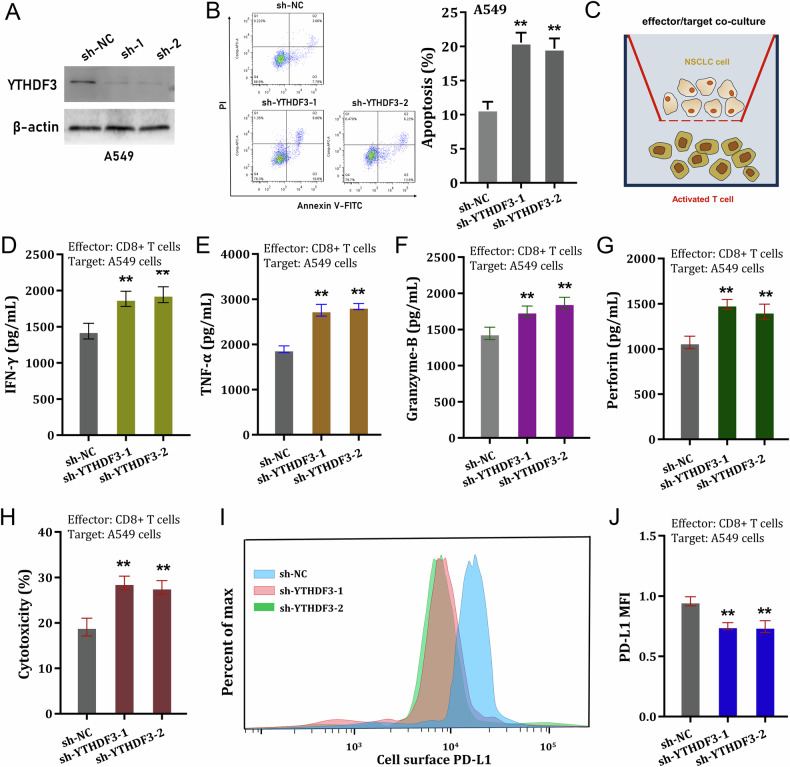
YTHDF3 silencing recovered CD8^+^ T antitumor activity to inhibit NSCLC immune evasion. **A** The YTHDF3 shRNAs were transfected into A549 cells and the silencing efficiency was detected using western blot. **B** The apoptosis of A549 cells were detected using flow cytometry. **C** The co-culture system was constructed using CD8^+^ T cells and NSCLC cells. **D** IFN-γ, **E** TNF-α, **F** Granzyme-B and **G** perforin secreted by CD8^+^ T cells were detected by ELISA kit upon co-cultured with YTHDF3 silencing transfected A549 cells. **H** The cytotoxicity was measured by LDH release assay after incubation with activated CD8^+^ T cells. **I**, **J** The cell-surface PD-L1 expression in NSCLC cells (A549) was detected by flow cytometry. *p < 0.05; **p < 0.01.

To test the function of YTHDF3 on NSCLC immune evasion, series of experiments were performed as following. Firstly, the YTHDF3 silencing was conducted in NSCLC cells (A549 cells) (Fig. 2A). In NSCLC cells, apoptosis analysis revealed that YTHDF3 silencing increased the apoptotic rate in A549 cells with shRNA transfection (Fig. 2B). To test the CD8^+^ T antitumor activity to NSCLC cells, co-culture system was constructed (Fig. 2C). NSCLC cells were incubated with activated CD8^+^ T cells for 48 h, and then the cytokines secreted by CD8^+^ T cells were detected, including IFN-γ, TNF-α, Granzyme-B and perforin. Results indicated that CD8^+^ T cells secreted significantly higher amounts of IFN-γ (Fig. 2D), TNF-α (Fig. 2E), Granzyme-B (Fig. 2F) and perforin (Fig. 2G) upon co-cultured with YTHDF3 silencing transfected A549 cells. After incubation with activated CD8^+^ T cells, the cytotoxicity was measured by LDH release assays, and the results indicated that CD8^+^ T cells exerted higher cytotoxicity activity upon co-cultured with YTHDF3 silencing transfected A549 cells (Fig. 2H). YTHDF3 silencing caused a significant lower expression of cell-surface PD-L1 expression in NSCLC cells (Fig. 2I, J). Overall, these findings revealed that YTHDF3 silencing recovered CD8^+^ T antitumor activity to inhibit NSCLC immune evasion.

### YTHDF3 upregulation impaired the CD8^+^ T antitumor activity to deteriorate immune evasion

**Fig. 3:**
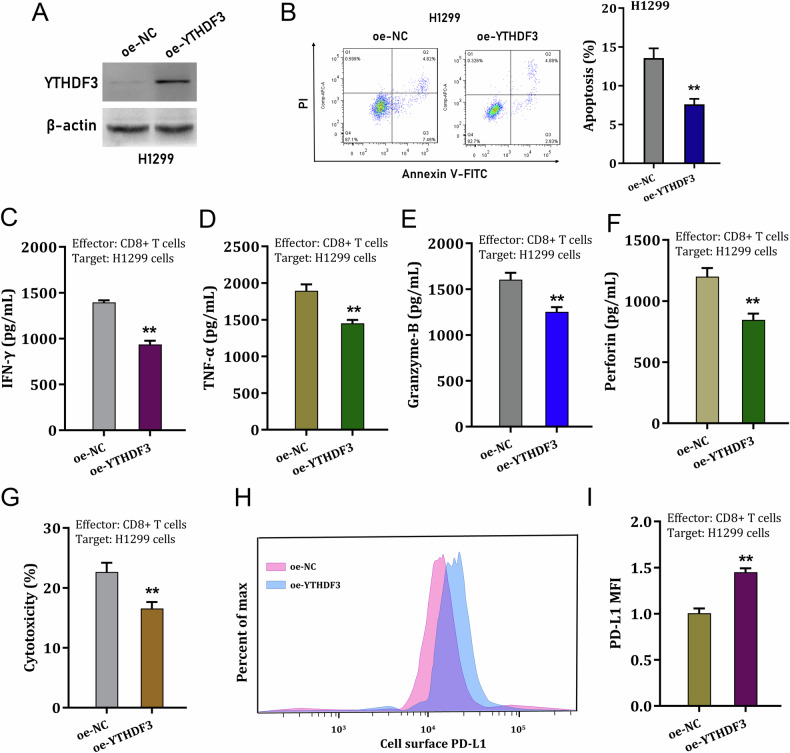
YTHDF3 up-regulation impaired the CD8^+^ T antitumor activity to deteriorate immune evasion. **A** The YTHDF3 overexpression plasmids were transfected into H1299 cells and the overexpression efficiency was detected using western blot. **B** The apoptosis of H1299 cells were detected using flow cytometry. **C** IFN-γ, **D** TNF-α, **E** Granzyme-B and **F** perforin secreted by CD8^+^ T cells were detected by ELISA kit upon co-cultured with YTHDF3 overexpression transfected H1299 cells. **G** The cytotoxicity was measured by LDH release assay after incubation with activated CD8^+^ T cells. **H**, **I** The cell-surface PD-L1 expression in NSCLC cells (H1299) was detected by flow cytometry. *p < 0.05; **p < 0.01.

Given YTHDF3 silencing recovered CD8^+^ T antitumor activity, the subsequent assays were performed to test the roles of YTHDF3 overexpression on NSCLC cells. The overexpression transfection of YTHDF3 was constructed in H1299 cells (Fig. 3A). Apoptosis analysis revealed that YTHDF3 overexpression reduced the apoptotic rate in H1299 cells with YTHDF3 overexpression transfection (Fig. 3B). In the co-culture system of CD8^+^ T and NSCLC cells, the cytokines secreted by CD8^+^ T cells were detected, including IFN-γ, TNF-α, Granzyme-B and perforin. Results indicated that CD8^+^ T cells secreted significantly lower amounts of IFN-γ (Fig. 3C), TNF-α (Fig. 3D), Granzyme-B (Fig. 3E) and perforin (Fig. 3F) upon co-cultured with YTHDF3 overexpression transfected H1299 cells. After incubation with activated CD8^+^ T cells, the cytotoxicity was measured by LDH release assays, and the results indicated that CD8^+^ T cells exerted lower cytotoxicity activity upon co-cultured with YTHDF3 overexpression transfected H1299 cells (Fig. 3G). Moreover, YTHDF3 overexpression caused a significant higher expression of cell-surface PD-L1 expression in NSCLC cells (Fig. 3H, I). Overall, these findings revealed that YTHDF3 up-regulation impaired the CD8^+^ T antitumor activity to deteriorate immune evasion.

### PD-L1 was the target of YTHDF3 via m^6^A-modified manner

**Fig. 4:**
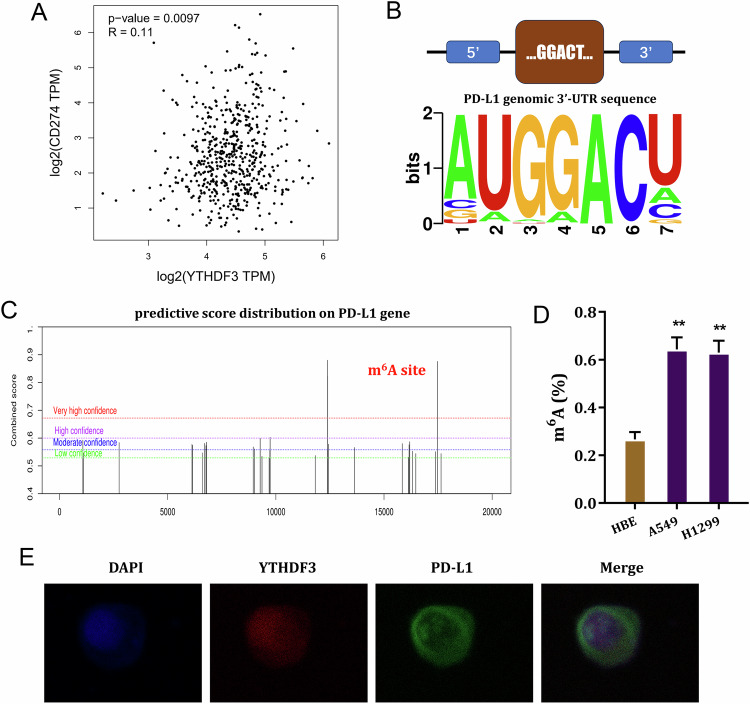
PD-L1 was the target of YTHDF3 via m^6^A-modified manner. **A** In clinical samples, the correlation within YTHDF3 and PD-L1 was analyzed by in silico tool (GEPIA, http://gepia.cancer-pku.cn/index.html). **B** The possible m^6^A motif towards YTHDF3 was identified as GGACU. **C** In silico predictive tool (SRAMP, http://www.cuilab.cn/sramp) indicated the several m^6^A modified sites on PD-L1gene. **D** The m^6^A modified level was detected in NSCLC cells (A549, H1299). **E** FISH assay indicated the co-location of YTHDF3 and PD-L1 in the A549 cells’ cytoplasm. **p < 0.01.

To test the depth mechanism by which YTHDF3 regulated NSCLC immune evasion, the potential downstream target of YTHDF3 was investigated. Firstly, in clinical samples, the correlation within YTHDF3 and PD-L1 exerted positive correlation (Fig. 4A). The possible m^6^A motif towards YTHDF3 was identified as GGACU (Fig. 4B). In silico predictive tool indicated that there were several m^6^A modified sites on PD-L1gene (Fig. 4C). In NSCLC cells, the m^6^A modified level was detected and results showed that m^6^A modification was higher in tumor cells (Fig. 4D). The sublocation of YTHDF3 and PD-L1 was detected using the FISH assay, and results indicated that YTHDF3 and PD-L1 were co-located in the A549 cells’ cytoplasm (Fig. 4E). Overall, these findings revealed that PD-L1 was the target of YTHDF3 via m^6^A-modified manner.

### YTHDF3 enhanced the PD-L1 mRNA stability

**Fig. 5:**
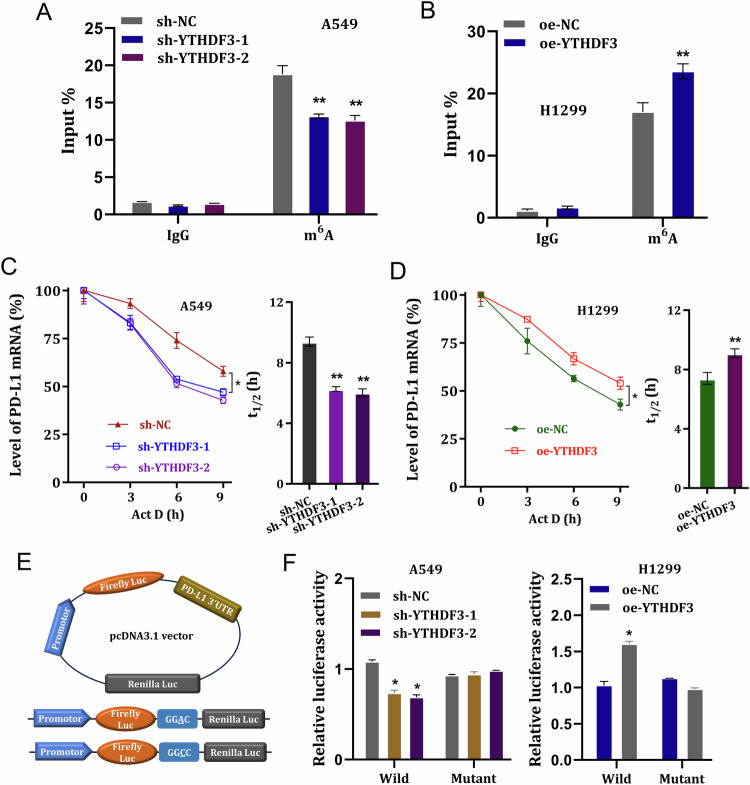
YTHDF3 enhanced the PD-L1 mRNA stability. **A**, **B** RIP assay using anti-m^6^A antibody was performed in NSCLC cells with YTHDF3 silencing (A) or YTHDF3 overexpression (B) to reflect the molecular interaction within YTHDF3 and PD-L1. **C**, **D** RNA stability analysis using RNA decay assay was performed to determine the half life time (t1/2) for PD-L1 mRNA with YTHDF3 silencing (A) or YTHDF3 overexpression (**D**). **E** The luciferase reporters with PD-L1 3′UTR wild-type (WT) sequences and corresponding mutant (Mut) with putative mutated m6A sites were constructed. **F** Luciferase activity assay was performed to detect the luciferase activity in A549 or H1299 cells with co-transfected with PD-L1-WT/Mutant plasmid. *p < 0.05; **p < 0.01.

To determine the function of YTHDF3 on PD-L1 mRNA’s fate, the depth mechanism assays were performed in this part. RIP assay using anti-m^6^A antibody revealed that YTHDF3 silencing reduced the molecular interaction within YTHDF3 and PD-L1 (Fig. 5A), and YTHDF3 overexpression enhanced the molecular interaction within YTHDF3 and PD-L1 (Fig. 5B). Then, the RNA stability analysis using RNA decay assay showed that YTHDF3 silencing reduced the half-life time for PD-L1 mRNA (Fig. 5C), and YTHDF3 overexpression enhanced the half-life time for PD-L1 mRNA (Fig. 5D). Then, luciferase reporters with PD-L1 3′UTR wild-type (WT) sequences and corresponding mutant (Mut) with putative mutated m6A sites were constructed (Fig. 5E). Luciferase activity assay indicated that YTHDF3 silencing reduced the luciferase activity with co-transfected with PD-L1-WT plasmid (Fig. 5F), and YTHDF3 overexpression enhanced it (Fig. 5G). Overall, these findings revealed that YTHDF3 enhanced the PD-L1 mRNA stability.

### YTHDF3 targeted PD-L1 to repress CD8^+^ T antitumor activity to induce NSCLC immune evasion

**Fig. 6:**
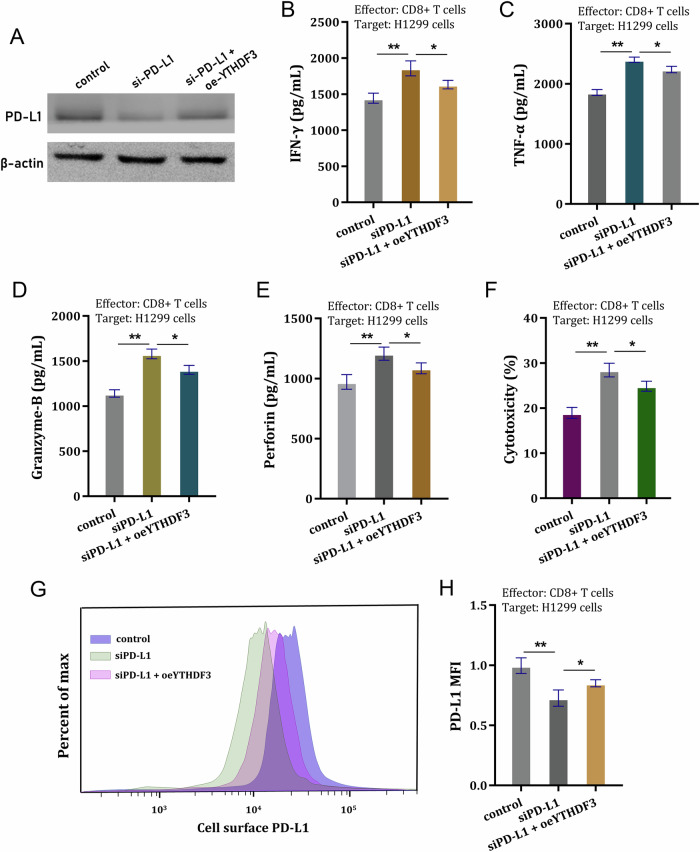
YTHDF3 targeted PD-L1 to repress CD8^+^ T antitumor activity to induce NSCLC immune evasion. **A** The YTHDF3 overexpression plasmids were transfected into H1299 cells and the overexpression efficiency was detected using western blot. (B-E) ELISA assays were performed to detect the (**B**) IFN-γ, (**C**) TNF-α, (**D**) Granzyme-B and (**E**) perforin secreted by CD8^+^ T cells co-cultured with H1299 cells transfected with YTHDF3 overexpression/si-PD-L1. **F** The cytotoxicity was measured by LDH release assay after incubation with activated CD8^+^ T cells. **G**, **H** The cell-surface PD-L1 expression in NSCLC cells (H1299) was detected by flow cytometry. *p < 0.05; **p < 0.01.

To further investigate the function of YTHDF3 on PD-L1-dependent CD8^+^ T antitumor activity, rescue assays were performed. Firstly, the YTHDF3 overexpression co-transfection could upregulate the PD-L1 protein level (Fig. 6A). In the co-culture system of CD8^+^ T and NSCLC cells, the cytokines secreted by CD8^+^ T cells were detected, including IFN-γ, TNF-α, Granzyme-B and perforin. Results indicated that CD8^+^ T cells secreted significantly lower amounts of IFN-γ (Fig. 6B), TNF-α (Fig. 6C), Granzyme-B (Fig. 6D) and perforin (Fig. 6E) upon co-cultured with PD-L1 siRNA (si-PD-L1) transfected H1299 cells. Besides, the YTHDF3 overexpression co-transfection could recover them. After incubation with activated CD8^+^ T cells, the cytotoxicity was measured by LDH release assays, and the results indicated that CD8^+^ T cells exerted lower cytotoxicity activity upon co-cultured with PD-L1 silencing (si-PD-L1) transfected NSCLC cells (H1299 cells) (Fig. 6F). Meanwhile, YTHDF3 overexpression co-transfection could recover it. Moreover, PD-L1 silencing (si-PD-L1) caused a significant lower expression of cell-surface PD-L1 expression in NSCLC cells (Fig. 6G, H), while YTHDF3 overexpression co-transfection could recover it. Overall, these findings revealed that YTHDF3 targeted PD-L1 to repress CD8^+^ T antitumor activity to induce NSCLC immune evasion.

### YTHDF3 silencing repressed the tumor growth and promoted the CD8^+^ T cells infiltration

**Fig. 7:**
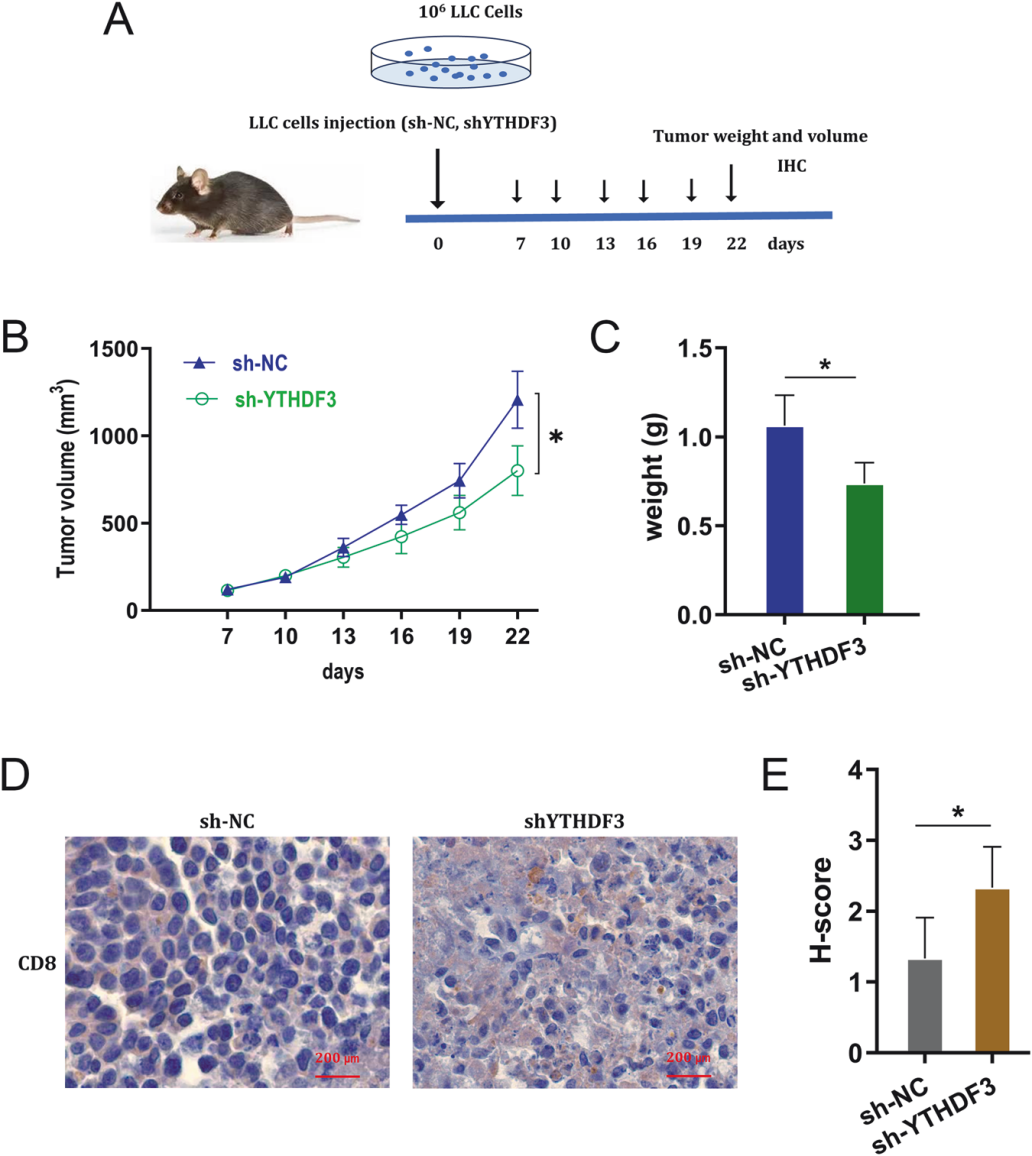
YTHDF3 silencing repressed the tumor growth and promoted the CD8^+^ T cells infiltration. **A** The in vivo assay was performed using the LLC cells in C57BL/6 mice. LLC cells were transfected by YTHDF3 silencing (sh-YTHDF3) or control (sh-NC). **B** The tumor volume and **C** weight were calculated. **D** The immumohistochemical staining of CD8 marker on CD8^+^ T cells in tumor tissue infiltration. **E** The H-score of the staining on CD8^+^ T cells.

To investigate the role of YTHDF3 on NSCLC immune evasion, the in vivo assay was performed using the LLC cells in C57BL/6 mice (Fig. 7A). The results demonstrated that YTHDF3 silencing repressed the tumor volume (Fig. 7B) and weight (Fig. 7C). Then, in the tumor tissue, the CD8^+^ T lymphocytes were marked by CD8 positive staining, and results indicated that YTHDF3 silencing group had higher CD8^+^ T cells infiltration (Fig. 7D, E). Therefore, the in vivo data indicated that YTHDF3 silencing repressed the tumor growth and promoted the CD8^+^ T cells infiltration.

## Discussion

Tumor immunotherapy is to mobilize the patient’s own immune system to kill tumor cells, so as to achieve the role of cancer treatment [14]. From the perspective of immunity response, cancer immunotherapy could restore or enhance the effector function of CD8^+^ T cells in the tumor microenvironment [15]. Thus, addressing the potential regulatory mechanism underlying NSCLC progression to develop new therapeutic strategies for NSCLC is of vital importance.

Given the critical function of m^6^A modification on tumor immune evasion [16, 17], this study performed series of assays to test the function of m^6^A reader YTHDF3 on NSCLC immune evasion. The result indicates that YTHDF3 was found to be highly expressed in NSCLC tissue and acted as an independent prognostic factor for overall survival. More and more studies have reported the correlation between m^6^A and tumor prognosis. For instance, m^6^A reader YTHDF2 expression is negatively correlated to HCC patients’ survival in both data from this study clinical data and Cancer Genome Atlas (TCGA) database [18]. Besides, m^6^A methyltransferase METTL3 acts as a marker for poor small cell lung cancer prognosis, and it is highly expressed in chemoresistant small cell lung cancer cells [19]. Thus, the key enzymes are closely correlated to lung cancer prognosis.

More literatures have indicated the critical function of CD8^+^ T cells on tumor immune response, including NSCLC [20, 21]. For instance, CD8^+^ T cells from NSCLC patients expressed an analogous gene expression program, which is distinct from conventional T cell exhaustion. CD8^+^ T cell differentiation limits the response of CD8^+^ T cells to immune checkpoint blockade, thereby contributing to immune checkpoint blockade failure in a subset T cell-infiltrated lung cancer [22]. In this research, our findings indicated that YTHDF3 up-regulation impaired the CD8^+^ T antitumor activity to deteriorate NSCLC immune evasion, while YTHDF3 silencing recovered the CD8^+^ T antitumor activity to inhibit immune evasion. Therefore, our findings suggested the function of CD8^+^ T cell on lung cancer.

Programmed death-ligand 1 (PD-L1) is a critical element for tumor immune escape microenvironment, which mediates the escape of tumor cells from CD8^+^ T cells’ killing. Low PD-L1 expression is more likely to experience better treatment benefit from anti-PD-1/PD-L1 agents (pembrolizumab, nivolumab, durvalumab, avelumab, atezolizumab) in advanced NSCLC. In NSCLC, the roles of PD-L1 have been widely reported through multitudinous mechanism [23]. PD-L1 could play a critical role as predictive biomarker for anti-PD-1/PD-L1 monotherapy with lower tumor PD-L1 expression to alternative treatments, such as combination immunotherapies. Higher PD-L1 expression in NSCLC is correlated to shorter survival in advanced/metastatic NSCLC, acting as a prognostic factor [24].

Here, in present research, results indicated that PD-L1 acted as the downstream target for YTHDF3, and YTHDF3 could upregulate the transcription stability of PD-L1 mRNA. With the assistance of YTHDF3, its downstream target’s fate could be modified via m^6^A-dependent manner. For example, YTHDF3 positively regulates NSCLC cells’ migration, invasion, and EMT in triple-negative breast cancer cells through enhancing ZEB1 mRNA stability in an m^6^A-dependent manner [25]. Overall, YTHDF3 targeted PD-L1 to promote NSCLC immune evasion partially through escaping effector cell cytotoxicity CD8^+^ T mediated killing and antitumor immunity.

**Fig. 8:**
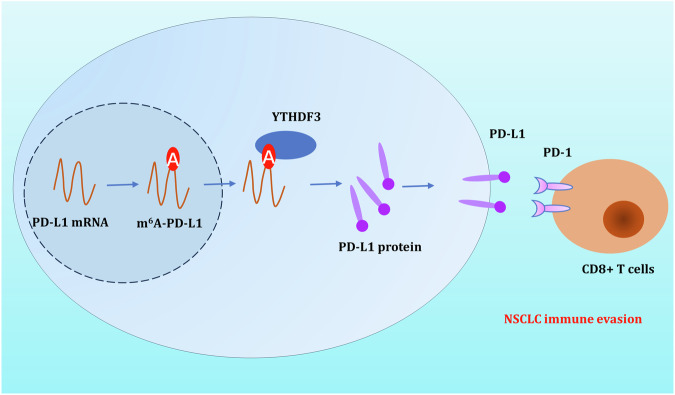
m^**6**^A reader YTHDF3 accelerates NSCLC immune evasion by inhibiting CD8^**+**^ T antitumor activity through m^**6**^A/PD-L1 manner.

In summary, the study suggests that YTHDF3 could accelerate the NSCLC immune evasion partially through repressing cytotoxicity CD8^+^ T mediated killing and antitumor immunity. The findings might provide an essential insight for epigenetics m^6^A modification and CD8^+^ T cell-mediated antitumor immunity in NSCLC (Fig. 8), which might provide a point for NSCLC immunotherapy.

## Materials and methods

### Clinical specimen collection

The clinical tissues, as well as their paired adjacent normal tissues, were collected from lung cancer patients who underwent surgical treatment in First Affiliated Hospital of Shenzhen University from 2020 to 2022. The clinicopathological characteristic of the cancer patients were listed in Table 1. All these clinical experiments in the present study had been approved by the Ethics Committee of First Affiliated Hospital of Shenzhen University. Besides, the written informed consents had been obtained from all the participants. The study was performed according to the ethical standards of the Declaration of Helsinki.

**Table 1:** Lung cancer clinicopathological feature for high or low YTHDF3 expression.

	Num	YTHDF3		*P* value
		Low (20)	High (20)	
Gender				0.333
Male	24	14	10	
Female	16	6	10	
Age (years)				0.525
<60	18	8	10	
≥60	22	12	10	
TNM				0.026*
I–II	21	14	7	
III/IV	19	6	13	
Differentiation				0.744
well/moderate	25	12	13	
poor	15	8	7	
Lymph metastasis				0.749
No	17	9	8	
Yes	23	11	12	

^*^p < 0.05 represents statistical difference.

### Cell lines, culture and vectors transfection

This study utilized human NSCLC cell lines (Calu-3, A549, SK-MES-1, H1299) and control cells (human normal bronchial epithelial cell, HBE) were provided from American Type Culture Collection (ATCC). Under standard condition (5% CO_2_, 37 °C), all the NSCLC cells and control cells were cultured in Roswell Park Memorial Institute 1640 (RPMI-1640) culture medium. To silence or enhance the indicated target, the vectors for YTHDF3 overexpression (YTHDF3-oe) and downregulation (YTHDF3-sh) were designed and manufactured by GenePharma (Shanghai, China). In addition, the PD-L1 inhibitor (si-PD-L1) and controls (si-NC) were designed and obtained from Ribobio (Guangzhou, China). The detailed transfection information was performed according to the above manufacture’s protocols.

### RNA isolation, qRT‑PCR, and western blot

Total RNA was isolated from NSCLC issue samples and cultured cell lines using TRIzol Reagent it (Invitrogen) according to the manufacturer’s protocols. RNA samples were reversely transcribed into cDNA by PrimeScript RT^TM^ Reagent Kit (TaKaRa) based on instruction. The qPCR was performed by SYBR Premix Taq (Applied Biosystems, US). The quantification of levels of mRNAs was performed by normalization to that of internal reference gene glyceraldehyde-3-phosphate dehydrogenase (GAPDH). The RNA relative expression levels (fold change) were analyzed by utilizing 2^−ΔΔCt^ methods. Primers sequences and oligonucleotides sequences were included in Additional File Supplementary Table S1. For western blot analysis, NSCLC cells were lysed utilizing radioimmunoprecipitation assay (RIPA) buffer (Yeasen, Shanghai, China). Cell lysate was centrifuged (15 min, 12,000 × *g*, 4 °C) and then the supernatants were collected. The collected protein was added to 10% SDS-PAGE gel and transferred to polyvinylidene fluoride (PVDF) membranes (Millipore, Billerica, MA, USA) by semidry blotters. Being blocked by 5% nonfat milk (1 h, room temperature), the membranes were incubated primary antibodies (anti-YTHDF3, 1:1000, Abcam, cat no. ab220161; anti-PD-L1, 1:1000, Abcam, cat no. ab213524) overnight at 4 °C. An GAPDH antibody (1:1000) served as the loading control. The protein amount was visualized by densitometry using enhanced chemiluminescence reagent.

### Immunohistochemistry staining and flow cytometry apoptosis analysis

For the tissue immunohistochemistry staining, cancer tissue paraffin sections were fixed by formalin and following deparaffinization and rehydration. For the incubation of primary antibody, anti-YTHDF3 (anti-YTHDF3, 1:1000 dilution, Abcam, cat no. ab220161) was incubated. The slide without primary antibody incubation acted as negative control. For the flow cytometry apoptosis analysis, the fluorescein isothiocyanate (FITC)-annexin V and propidium iodide (PI) was utilized for double staining with FITC Annexin V Apoptosis Detection Kit (BD Biosciences) according to the manufacturer’s recommendation.

### CD8^+^ T cell isolation and co-culture system

CD8^+^ T cells were obtained from healthy volunteer donors’ peripheral blood mononuclear cells (PBMCs). The PBMCs were isolated and purified using Easy-Sep™ Direct Human CD8^+^ T Cell Isolation Kit (STEMCELL Technologies, Vancouver, Canada, US, Cat no. #15063). For the activation, human CD8^+^ T cells were seeded into 24-well plates and induced with anti-CD3/anti-CD28 antibodies (2 µl/well) and IL-2 (20 ng/mL) (BD Biosciences, Franklin Lakes, NJ, USA) and incubated for 48 h. For the in vitro co-culture system, the activated CD8^+^ T cells were co-cultured with adhered cancer cells at 5:1 ratio (effector to target) for 48 h.

### Enzyme‑linked immunosorbent assay (ELISA)

The ELISA assay was performed to detect the concentrations CD8^+^ T cells secreted cytokines, including IFN-γ, TNF-α, Granzyme-B and Perforin with commercial kit in accordance with the manufacturer’s guideline. The kits were following: IFN-γ, BD Pharmingen, cat no. 550583; TNF-α, eBioscience, cat no. BMS223HS; Granzyme-B, eBioscience, cat no. BMS2027; Perforin, eBioscience, cat no. BMS2306.

### Cytotoxicity assay

The CD8^+^ T cell-mediated cytotoxicity on NSCLC cells was determined by lactate dehydrogenase (LDH)-based cytotoxicity assay. After co-culture of NSCLC cells and CD8+ T cells, the culture supernatants were collected for LDH release using Cytotoxicity Detection Kit PLUS (Sigma-Aldrich, catalog no. 04744926001) according to the manufacturer’s recommendation.

### Surface PD-L1 expression analysis

To measure cellular surface PD-L1 expression, NSCLC cells were resuspended in PBS buffer, and incubated with primary anti-PD-L1 antibodies (BioLegend) according to standard protocols for flow cytometry. Consequently, flow cytometric data were analyzed using the FlowJo software program.

### m^6^A modified level

The total RNA was extracted from NSCLC cells using TRIzol reagent kit (Invitrogen). RNA sample (200 ng) were added into 96-well plates and each well was added with diluted capture antibody and diluted enhancer solution (100 mL). The termination solution was supplemented to each well on the microplate reader at 450 nm. The m^6^A level in total RNA was detected using an m6A RNA methylation detection kit (cat no. ab185912, Abcam).

### Fluorescence in situ hybridization (FISH)

FISH was performed using an mRNA in situ hybridization kit (Shanghai GenePharma Co., Ltd, China). NSCLC cells samples were incubated with probe buffer overnight at 37 °C in a dark moist chamber. After being washed twice in 50% formamide for 5 min, the slices were incubated with the regents and sealed with DAPI parafilm. Labeled cells were imaged by immunofluorescence under a confocal fluorescence microscopy (OLYMPUS FV1000 confocal microscopy, Japan).

### RNA immunoprecipitation

RNA immunoprecipitation (RIP) experiments were performed using Magna RIP RNA-Binding Protein Immunoprecipitation Kit (Millipore, Billerica, MA, USA) based on the manufacturer’s recommendation. Briefly, 5 × 10^6^ cells were collected and resuspended in RIPA lysis buffer (300 μL) with RNase inhibitor and protease inhibitor. The cell lysates (200 μL) were incubated with control IgG antibody or anti-YTHDF3 (1:1000, Abcam, cat no. ab220161) or with A/G magnetic beads overnight at 4 °C. The immunoprecipitated RNA was purified and evaluated by qPCR.

### Luciferase reporter assay

For firefly luciferase construction, cDNAs containing PD-L1 genome 3′-UTR sequence were cloned into pGL3-control vectors (Promega). Besides, cytosine (C) was replaced to marked adenosine (A) in m6A motif for mutant. NSCLC cells were co-transfected with PD-L1 wild-type (0.5 μg) or mutated reporter plasmids (25 ng, renilla luciferase reporter vector). After 24 h, cells were harvested and the luciferase activity was detected using Dual-Glo Luciferase system (Promega). The luciferase activity was normalized to Renilla activity.

### RNA stability assay

NSCLC cells were seeded in the 6-well plates and incubated overnight. At indicated time, NSCLC cells were treated with actinomycin D (5 μg/mL, MedChemExpress, Cat no. HY-17559) for 0, 3, 6, 9 h. Total RNA was isolated from NSCLC cells using TRIzol kit and relative level was quantified by qRT-PCR, which was calculated and normalized by GAPDH.

### In vivo animal assay

C57BL/6 mice were provided from SLAC (Shanghai, China) and then housed in pathogen-free condition. Mice were inoculated with total of 10^6^ LLC cells that transfected sh-NC or sh-YTHDF3. The volume and weight were calculated as protocols. This assay was approved by Ethics Committee of First Affiliated Hospital of Shenzhen University.

### Statistical analysis

All experiments were performed via three independent assays, which was shown as mean ± standard deviation (SD). Statistic was assessed using Student’s *t*-test or one-way analysis of variance (ANOVA) following Tukey’s post-hoc tests. Data analysis was performed using GraphPad Prism software version 9.0. and SPSS software version 22.0. *p* < 0.05 was supposed to statistically significant.

### Supplementary information

SUPPLEMENTAL MATERIAL

## Data Availability

The datasets generated during and/or analyzed during the current study are available from the corresponding author on reasonable request.

## References

[1] Alexander M, Kim SY, Cheng H (2020). Update 2020: management of non-small cell lung cancer. Lung.

[2] Duma N, Santana-Davila R, Molina JR (2019). Non-small cell lung cancer: epidemiology, screening, diagnosis, and treatment. Mayo Clin Proc.

[3] Ettinger DS, Wood DE, Aisner DL, Akerley W, Bauman JR, Bharat A (2022). Non-small cell lung cancer, version 3.2022, NCCN clinical practice guidelines in oncology. J Natl Compr Cancer Netw.

[4] Herbst RS, Morgensztern D, Boshoff C (2018). The biology and management of non-small cell lung cancer. Nature.

[5] Liu Z, Wang T, She Y, Wu K, Gu S, Li L (2021). N(6)-methyladenosine-modified circIGF2BP3 inhibits CD8(+) T-cell responses to facilitate tumor immune evasion by promoting the deubiquitination of PD-L1 in non-small cell lung cancer. Mol Cancer.

[6] Mithoowani H, Febbraro M (2022). Non-small-cell lung cancer in 2022: a review for general practitioners in oncology. Curr Oncol.

[7] Reck M, Remon J, Hellmann MD (2022). First-line immunotherapy for non-small-cell lung cancer. J Clin Oncol.

[8] Hong W, Xue M, Jiang J, Zhang Y, Gao X (2020). Circular RNA circ-CPA4/let-7 miRNA/PD-L1 axis regulates cell growth, stemness, drug resistance and immune evasion in non-small cell lung cancer (NSCLC). J Exp Clin Cancer Res.

[9] Chen X, Gao A, Zhang F, Yang Z, Wang S, Fang Y (2021). ILT4 inhibition prevents TAM- and dysfunctional T cell-mediated immunosuppression and enhances the efficacy of anti-PD-L1 therapy in NSCLC with EGFR activation. Theranostics.

[10] Bagchi S, Yuan R, Engleman EG (2021). Immune checkpoint inhibitors for the treatment of cancer: clinical impact and mechanisms of response and resistance. Annu Rev Pathol.

[11] Li W, Wu F, Zhao S, Shi P, Wang S, Cui D (2022). Correlation between PD-1/PD-L1 expression and polarization in tumor-associated macrophages: A key player in tumor immunotherapy. Cytokine Growth Factor Rev.

[12] Bassanelli M, Sioletic S, Martini M, Giacinti S, Viterbo A, Staddon A (2018). Heterogeneity of PD-L1 expression and relationship with biology of NSCLC. Anticancer Res.

[13] Brody R, Zhang Y, Ballas M, Siddiqui MK, Gupta P, Barker C (2017). PD-L1 expression in advanced NSCLC: Insights into risk stratification and treatment selection from a systematic literature review. Lung Cancer.

[14] Zhang B, Wu Q, Li B, Wang D, Wang L, Zhou YL (2020). m(6)A regulator-mediated methylation modification patterns and tumor microenvironment infiltration characterization in gastric cancer. Mol Cancer.

[15] Sivori S, Pende D, Quatrini L, Pietra G, Della Chiesa M, Vacca P (2021). NK cells and ILCs in tumor immunotherapy. Mol Asp Med.

[16] Zhao W, Liu J, Wu J, Ma X, Wang X, Zhang L (2022). High-throughput microarray reveals the epitranscriptome-wide landscape of m(6)A-modified circRNA in oral squamous cell carcinoma. BMC Genomics.

[17] Xiong J, He J, Zhu J, Pan J, Liao W, Ye H (2022). Lactylation-driven METTL3-mediated RNA m(6)A modification promotes immunosuppression of tumor-infiltrating myeloid cells. Mol Cell.

[18] Zhang C, Huang S, Zhuang H, Ruan S, Zhou Z, Huang K (2020). YTHDF2 promotes the liver cancer stem cell phenotype and cancer metastasis by regulating OCT4 expression via m6A RNA methylation. Oncogene.

[19] Sun Y, Shen W, Hu S, Lyu Q, Wang Q, Wei T (2023). METTL3 promotes chemoresistance in small cell lung cancer by inducing mitophagy. J Exp Clin Cancer Res..

[20] Papa-Gobbi R, Muglia CI, Rocca A, Curciarello R, Sambuelli AM, Yantorno M (2021). Spatiotemporal regulation of galectin-1-induced T-cell death in lamina propria from Crohn’s disease and ulcerative colitis patients. Apoptosis.

[21] Wang Y, Jin P, Wang X. N(6)-methyladenosine regulator YTHDF1 represses the CD8+ T cell-mediated antitumor immunity and ferroptosis in prostate cancer via m(6)A/PD-L1 manner. Apoptosis. 2024;29:142–153. 10.1007/s10495-023-01885-7 37698736

[22] Horton BL, Morgan DM, Momin N, Zagorulya M, Torres-Mejia E, Bhandarkar V (2021). Lack of CD8(+) T cell effector differentiation during priming mediates checkpoint blockade resistance in non-small cell lung cancer. Sci Immunol.

[23] Eguren-Santamaria I, Sanmamed MF, Goldberg SB, Kluger HM, Idoate MA, Lu BY (2020). PD-1/PD-L1 blockers in NSCLC brain metastases: challenging paradigms and clinical practice. Clin Cancer Res.

[24] Wang J, Xu Y, Rao X, Zhang R, Tang J, Zhang D (2022). BRD4-IRF1 axis regulates chemoradiotherapy-induced PD-L1 expression and immune evasion in non-small cell lung cancer. Clin Transl Med.

[25] Lin Y, Jin X, Nie Q, Chen M, Guo W, Chen L (2022). YTHDF3 facilitates triple-negative breast cancer progression and metastasis by stabilizing ZEB1 mRNA in an m(6)A-dependent manner. Ann Transl Med.

