# Supplementary material for: YTH domain family protein 3 accelerates non-small cell lung cancer immune evasion through targeting CD8+ T lymphocytes
Source: Cell Death Discov. 2024 Jul 11;10:320. doi: 10.1038/s41420-024-02084-2 (PMC11239943; doi:10.1038/s41420-024-02084-2)
Supplement: Supplementary file 1 — SUPPLEMENTAL MATERIAL [file 41420_2024_2084_MOESM1_ESM.pdf]

Fig 2 and fig 3   YTHDF3

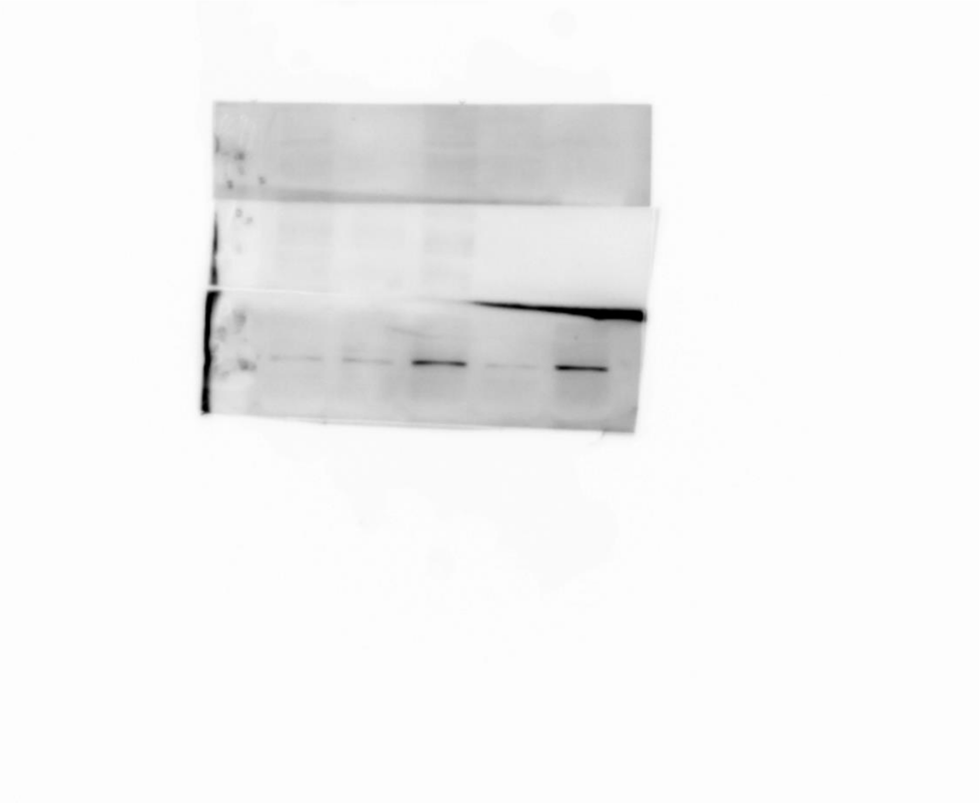

Fig 2 and fig 3   actin

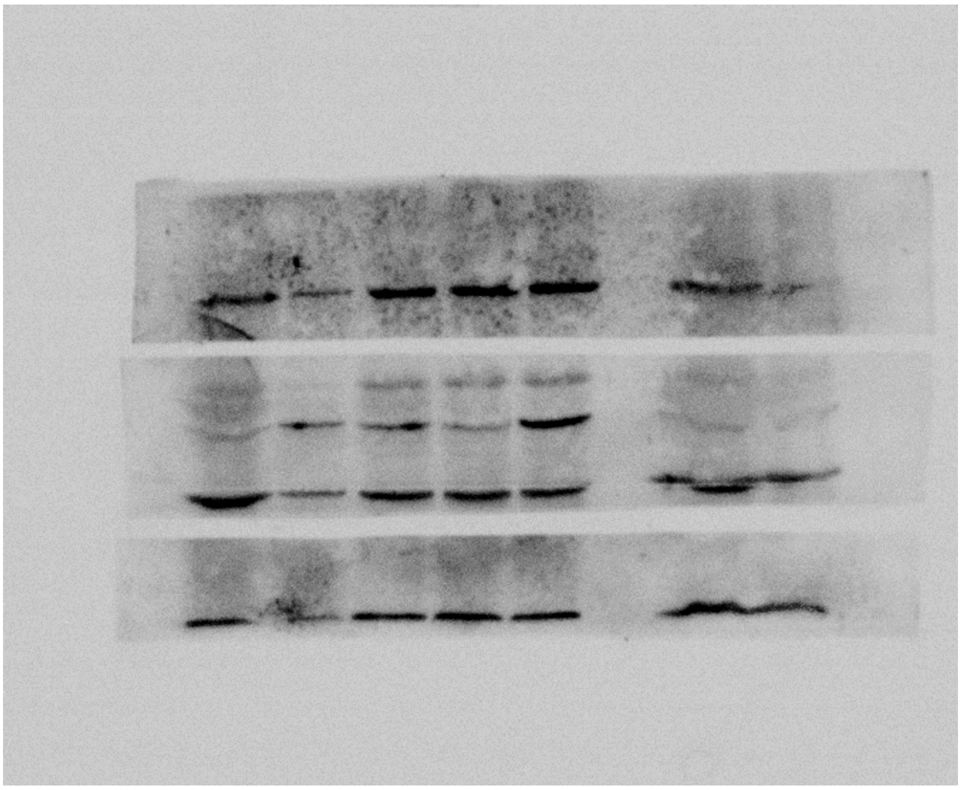

supplementary Table S1. qRT-PCR primers sequences and shRNA sequences.

	Sequences
YTHDF3	forward, 5'- GGTGTATTTAGTCAACCTGGGG-3'
	reverse, 5'- AAGAGAACTAGGTGGATAGCCAT-3'
PD-L1	forward, 5’- GCTGCACTAATTGTCTATTGGGA-3’
	reverse, 5’- AATTCGCTTGTAGTCGGCACC-3’
sh-YTHDF3-1	5'- CCAACTTCTTGGGCTGCTATT-3'
sh-YTHDF3-2	5'- CCAATAACCAATTACGACATA-3'
beta-actin	forward, 5’-CTCCATCCTGGCCTCGCTGT-3’
	reverse, 5’-GCTGTCACCTTCACCGTTCC-3’
